# The Role of PTP1B *O-*GlcNAcylation in Hepatic Insulin Resistance

**DOI:** 10.3390/ijms160922856

**Published:** 2015-09-22

**Authors:** Yun Zhao, Zhuqi Tang, Aiguo Shen, Tao Tao, Chunhua Wan, Xiaohui Zhu, Jieru Huang, Wanlu Zhang, Nana Xia, Suxin Wang, Shiwei Cui, Dongmei Zhang

**Affiliations:** 1 Department of Endocrinology, Affiliated Hospital of Nantong University, Nantong 226001, China; E-Mails: zhaoyunnt@163.com (Y.Z.); tangzhuqi@aliyun.com (Z.T.); neizhuhui@163.com (X.Z.); liangchenyinuan@126.com (J.H.); wang496369@163.com (W.Z.); 15851209084@163.com (N.X.); wangsuxin2009@sina.com (S.W.); 2 Jiangsu Province Key Laboratory for Inflammation and Molecular Drug Target, Nantong University, Nantong 226001, China; E-Mails: shag@ntu.edu.cn (A.S.); nttaotao@ntu.edu.cn (T.T.); zhao1027601171@163.com (C.W.); 3 Co-Innovation Center of Neuroregeneration, Nantong University, Nantong 226001, China

**Keywords:** insulin resistance, PTP1B, *O*-GlcNAc

## Abstract

Protein tyrosine phosphatase 1B (PTP1B), which can directly dephosphorylate both the insulin receptor and insulin receptor substrate 1 (IRS-1), thereby terminating insulin signaling, reportedly plays an important role in insulin resistance. Accumulating evidence has demonstrated that *O-*GlcNAc modification regulates functions of several important components of insulin signal pathway. In this study, we identified that PTP1B is modified by *O*-GlcNAcylation at three *O-*GlcNAc sites (Ser104, Ser201, and Ser386). Palmitate acid (PA) impaired the insulin signaling, indicated by decreased phosphorylation of both serine/threonine-protein kinase B (Akt) and glycogen synthase kinase 3 beta (GSK3β) following insulin administration, and upregulated PTP1B *O-*GlcNAcylation in HepG2 cells. Compared with the wild-type, intervention PTP1B *O*-GlcNAcylation by site-directed gene mutation inhibited PTP1B phosphatase activity, resulted in a higher level of phosphorylated Akt and GSK3β, recovered insulin sensitivity, and improved lipid deposition in HepG2 cells. Taken together, our research showed that *O*-GlcNAcylation of PTP1B can influence insulin signal transduction by modulating its own phosphatase activity, which participates in the process of hepatic insulin resistance.

## 1. Introduction

Insulin resistance, a state in which peripheral tissues demonstrate a diminished response to the glucose-lowering properties of insulin, plays a central role in the development of type 2 diabetes mellitus (T2DM). After insulin stimulation, insulin receptor (IR) is autophosphorylated. Activated IR phosphorylates the downstream docking protein insulin receptor substrate 1 (IRS-1), which, subsequently, through the activation of the phosphatidylinositol 3-kinase (PI3K) and Akt/protein kinase B (PKB) pathway, leads to the translocation of glucose transporter type 4 (GLUT4) vesicles to the cell surface and triggers cellular glucose uptake [1]. Insulin resistance is characterized by impaired peripheral glucose uptake, increased hepatic glucose production and abnormal lipid deposition [2].

The involvement of protein tyrosine phosphatase 1B (PTP1B) in insulin signaling has been suggested in numerous reports [3,4,5,6]. PTP1B possesses a catalytic domain characterized by the 11 amino acid sequence motif: (I/V)HCXAGXXR(S/T)G. This motif contains cysteine (Cys215) and arginine (Arg221) residues critical for the catalytic activity of the enzyme [7]. The conserved phosphatase domain of PTP1B is contained within the domain spanning residues 30 to 278. The COOH-terminal non-catalytic extension of the protein serves a regulatory function. The COOH-terminal 35 residues target the enzyme to the cytoplasmic face of the endoplasmic reticulum [8]. As an important phosphatase, PTP1B inhibits insulin-stimulated IR autophosphorylation and phosphorylation of IRS proteins. This dephosphorylation inactivates the IR and thereby terminates the insulin signaling [9,10]. Specifically targeting the liver, PTP1B appears to be an attractive drug therapy for the treatment of metabolic syndrome, as it not only improves whole-body insulin sensitivity but also decreases lipid deposition in the liver [11,12]. Whole-body PTP1B knockout mice exhibit improved systemic insulin sensitivity and enhanced glucose tolerance, and are resistant to the obesity induced by a high fat diet [13]. These data suggest that PTP1B acts as an important negative regulator of insulin signaling.

Glucose flux through the hexosamine biosynthetic pathway (HBP) leads to the post-translational modification of cytoplasmic and nuclear proteins by *O*-linked-*N*-acetylglucosamine (*O*-GlcNAc). *O*-GlcNAc rapidly cycles on serine and threonine residues in a fashion analogous to phosphorylation. Unlike phosphorylation, the addition of *O*-GlcNAc is performed by a single catalytic subunit (*O*-GlcNAc transferase, OGT), and the removal is achieved by *O*-GlcNAc selective *N*-acetyl-β-d-glucosaminidase (*O*-GlcNAcase, OGA). Emerging evidence suggests *O*-GlcNAc’s putative roles in the pathogenesis of insulin resistance. Increased intracellular protein *O*-GlcNAc modification has been observed in diabetes and hyperglycemic states, like FoxO1, ChREBP, CRTC2 *et al.* [14,15,16,17]. OGT attenuates insulin signaling by *O*-GlcNAcylation of proteins involved in proximal and distal steps in the PI-3 kinase signaling pathway [18,19]. In addition, O-GlcNAc directly contributes to diabetic complications [20,21,22].

Diverse post-translational modifications have been reported to regulate PTP1B functions, including oxidation, nitrosylation, sulfyhydration, and phosphorylation [6]; however, whether PTP1B can be *O*-GlcNAc modified remains unclear. In this study, we observed that PTP1B has three *O*-GlcNAc sites: Ser104, Ser201, Ser386; *O*-GlcNAcylation of PTP1B is increased in insulin resistance; and intervention PTP1B *O*-GlcNAcylation can recover insulin sensitivity and improve lipid deposition in HepG2 cells. This study helps us to further understand the contribution of post-translational modification of PTP1B to the etiology of insulin resistance.

## 2. Results and Discussion

### 2.1. PTP1B Is Modified by O-GlcNAcylation

**Figure 1:**
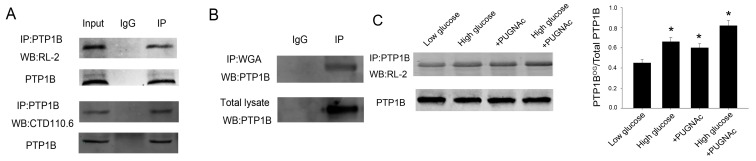
PTP1B is modified by *O*-GlcNAcylation. (**A**) To detect whether PTP1B is *O*-GlcNAcylated, PTP1B was immunoprecipitated from 500 μL lysate of HepG2 cells with 5 μL PTP1B antibody and analyzed by western blot with RL-2 and CTD110.6 antibody. Input was used as positive control. IgG was a negative control; (**B**) Cell lysate was immunoprecipitated with WGA agarose beads followed by PTP1B antibody; (**C**) HepG2 cells were incubated for 24 h under low (5 mmol/L) or high (25 mmol/L) glucose conditions and treated with or without the O-GlcNAcase inhibitor PUGNAc. Then PTP1B was immunoprecipitated and analyzed by western blot with RL-2 antibody. IP, immunoprecipitation; WB, western blot. Data show mean ± SEM of three independent experiments. (*n* = 3, * *p* < 0.05, significantly different from respective controls).

Given that PTP1B plays an important role in insulin resistance and *O*-GlcNAc functioned as a nutrient sensor, we tested whether PTP1B was *O*-GlcNAc modified in HepG2 cells. Figure 1 showed that PTP1B was *O*-GlcNAcylated using the anti-*O*-GlcNAc antibodies, RL-2 and CTD110.6 (Figure 1A), and the terminal GlcNAc-specific lectin, succinylated wheat germ agglutinin (WGA) (Figure 1B). Then we treated HepG2 cells with 5 mmol/L (low glucose, LG) or 25 mmol/L (high glucose, HG) for 24 h, monitoring the increase of *O*-GlcNAc modification under the high glucose condition, which imitated a diabetic glucose concentration. PTP1B *O*-GlcNAcylation increased under HG conditions compared with LG conditions based on immunoprecipitation with an anti-PTP1B antibody followed by immunoblotting with an anti-RL-2 antibody (Figure 1C). In line with the results, following treatment of the cells with the *O*-GlcNAcase inhibitor (PUGNAc), another approach to increase protein *O*-GlcNAc concentrations, we also observed increased *O*-GlcNAc levels of PTP1B, which further confirmed that PTP1B was an *O*-GlcNAc modified protein (Figure 1C).

### 2.2. O-GlcNAcylation of PTP1B Is Increased in Insulin Resistance

**Figure 2:**
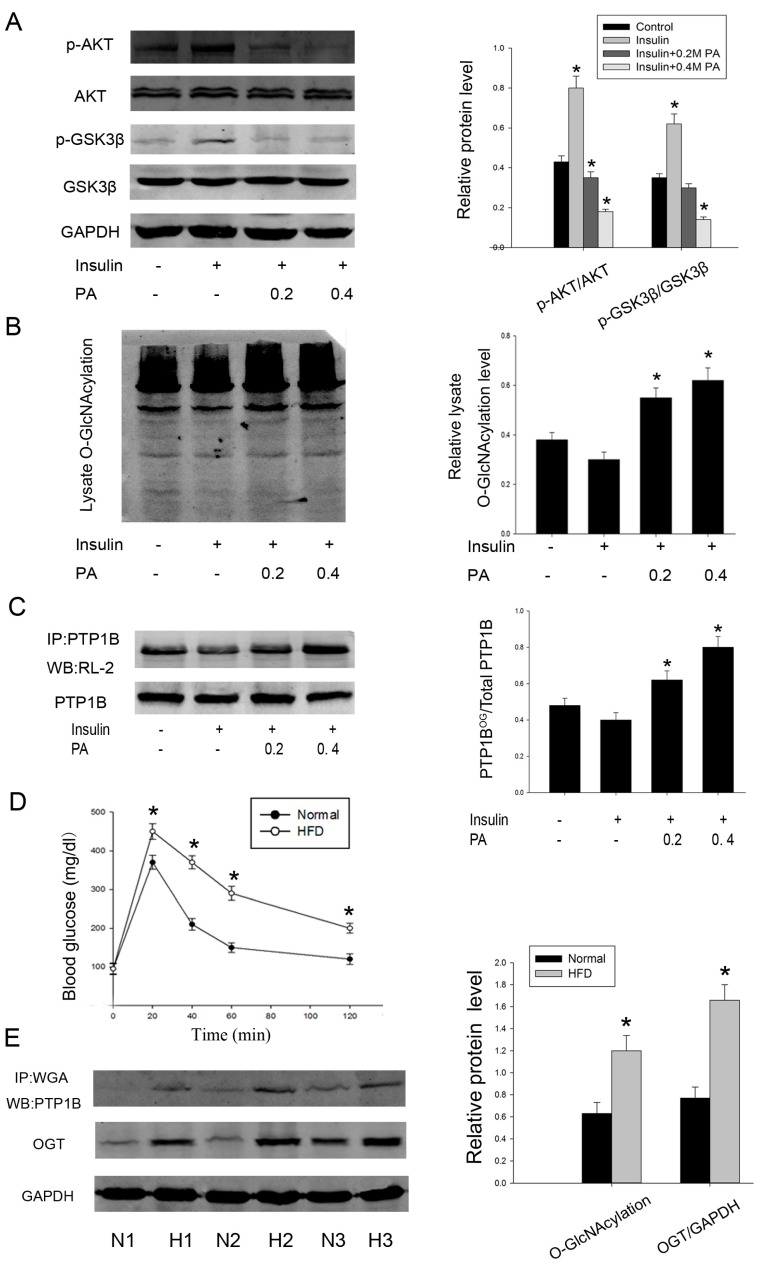
*O*-GlcNAcylation of PTP1B is increased in insulin resistance. Human HepG2 cells were treated with palmitate (0/0.2/0.4 mmol/L) for 24 h before being stimulated with insulin for 20 min. (**A**) Proteins were extracted and the phosphorylation of Akt and GSK3β was measured by western blot analysis; (**B**) *O*-GlcNAcylation of total cell lysate was analyzed; (**C**) *O*-GlcNAcylation of PTP1B was evaluated by immunoprecipitation followed by western blot analysis; (**D**) mice insulin resistance model and glucose tolerance test; (**E**) hepatic tissues were obtained from three groups of mice fed a normal or a high fat diet, respectively. *O*-GlcNAcylation of PTP1B and expression of OGT were measured. Similar results were obtained in two other experiments. (*n* = 3, * *p* < 0.05, significantly different from respective controls).

Chronic elevation in plasma free fatty acid (FFA) level is commonly associated with obesity and T2DM. Palmitate acid (PA) is one form of saturated FFAs, which are frequently used to induce insulin resistance [23]. So we first determined the effects of palmitate on insulin sensitivity. HepG2 cells were incubated for 24 h in the presence of increasing concentrations of palmitate (0, 0.2, and 0.4 mmol/L), and insulin was added during the last 20 min of incubation. The phosphorylation of Akt (Ser473) and GSK3β (Ser21/9), previously reported to mediate the activation of insulin signaling, was analyzed. As shown in Figure 2A, palmitate strongly decreased phosphorylation of these two proteins when compared with only insulin-stimulated cells, which suggesting an impaired sensitivity to insulin. Moreover, a palmitate concentration of 0.4 mmol/L was more effective, but higher concentrations (0.6 or 0.8 mmol/L) were inappropriate because higher concentrations of palmitate over 24 h incubation could induce cell apoptosis.

We next asked whether intracellular *O*-GlcNAc level was altered in this cell model of insulin resistance. We treated HepG2 cells as previously described and found that *O*-GlcNAcylation of total protein was increased by palmitate (Figure 2B). Concurrent with this result, palmitate also increased the *O*-GlcNAcylation level of PTP1B immunoprecipitates (Figure 2C). Interestingly, the addition of 100 nM insulin for 20 minutes upregulated phosphorylation of Akt and GSK3β, whereas it downregulated total cellular *O*-GlcNAc levels and *O*-GlcNAcylation, specifically on PTP1B. These data suggest that there exists a potential correlation between a fall in insulin-stimulated phosphorylation of signaling components and an increase in PTP1B *O*-GlcNAcylation.

To further identify the increased level of PTP1B *O*-GlcNAcylation in insulin resistance, we then examined the *O*-GlcNAc level in normal and insulin-resistant liver tissues from mice fed either a normal or a high fat diet. Glucose tolerance test was conducted after 12 weeks and confirmed the insulin resistance model (Figure 2D). Consistently, *O*-GlcNAcylation of PTP1B revealed by WGA was also markedly increased in high fat diet mice. Because OGT was required for the addition of *O*-GlcNAc, we also measured the expression of OGT. Similarly, OGT expression was increased by about two-fold when compared with normal diet mice (Figure 2E). Taken together, these results indicate that there is a close correlation between increased PTP1B *O*-GlcNAcylation and occurrence of insulin resistance.

### 2.3. PTP1B Has Three O-GlcNAc Sites and O-GlcNAcylation Influences Its Phosphatase Activity

**Figure 3:**
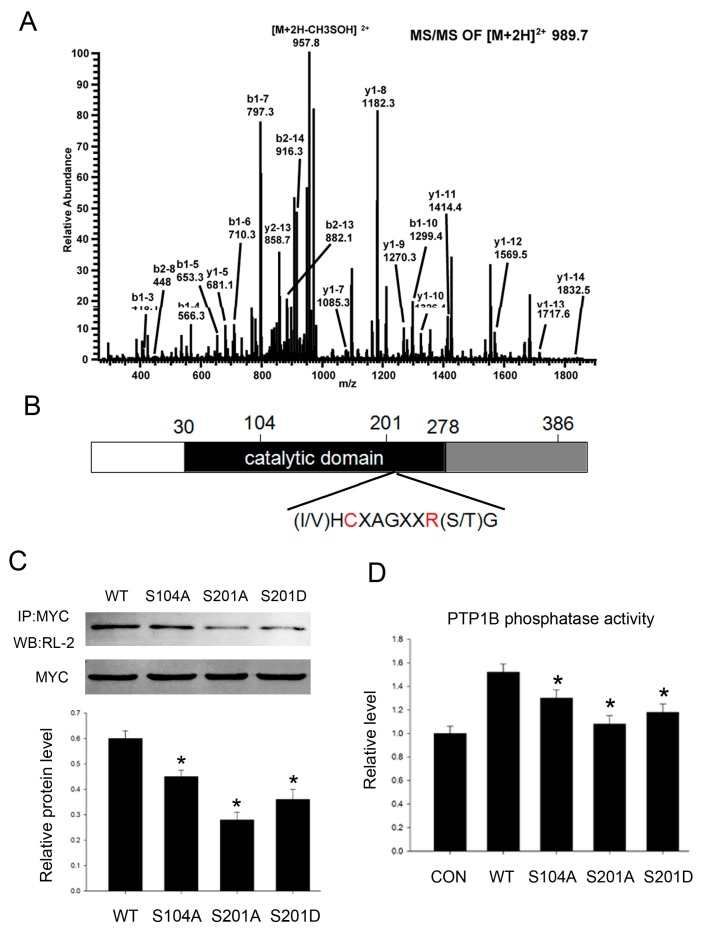
PTP1B is *O*-GlcNAc modified at three sites. (**A**) ETD MS/MS site mapping of the human PTP1B *O*-GlcNAc modification sites; (**B**) Schematic diagram shows full-length PTP1B domain structure and *O*-GlcNAc sites (Ser104, Ser201, and Ser386); C (Cys215) and A (Arg221), two residues which are critical for the catalytic activity of PTP1B; (**C**) We constructed site-directed mutants (Ser104 mutated to alanine, Ser201 mutated to alanine and aspartic acid), then HepG2 cells were transfected with pcDNA3.1/myc-His (−) vectors containing the indicated site mutations for 24 h before treating with 0.2 mM palmitate acid for 18 h. Then cells were immunoprecipitated with an anti-myc antibody followed by immunoblotting with an anti-*O*-GlcNAc antibody (RL-2); (**D**) PTP1B phosphatase activity was analyzed by protein phosphatase assay kit according to the manufacturer’s instruction. Data show mean ± SEM of three independent experiments. (*n* = 3, * *p* < 0.05, significantly different from respective controls).

Because PTP1B acts as an important phosphatase, the enzyme activity is crucial to its functions. To better understand how *O*-GlcNAc regulates PTP1B phosphatase activity and its role in insulin resistance, we conducted mass spectrometry studies to characterize *O*-GlcNAc sites in PTP1B and identified three residues: Ser104, Ser201, and Ser386 (Figure 3A), two of which (Ser104 and Ser201) are within the conserved phosphatase catalytic domain (Figure 3B). So we supposed that *O*-GlcNAcylation at these two sites might be important to its phosphatase activity. Simultaneously, it should be noted that Ser201 and Ser386 are also phosphorylation sites.

Next we conducted site-directed mutants containing Ala substitution at Ser104 (S104A) and Ser201 (S201A) to block *O*-GlcNAcylation at these sites, and aspartate substitution at Ser201 to mimic constitutive phosphorylation at this site (S201D). Then we detected PTP1B *O*-GlcNAcylation level when *O*-GlcNAc sites were mutated. All three mutants showed lower amounts of *O*-GlcNAcylation when compared with the wild-type, further confirming they are *O*-GlcNAc sites (Figure 3C).

Next we tested how *O*-GlcNAcylation regulated PTP1B phosphatase activity. We found site-directed mutant PTP1B phosphatase activity was impaired when compared with the wild-type, and the level of its phosphatase activity was concomitant with its *O*-GlcNAcylation level. Mutation at Ser201 was more significant. Constitutive phosphorylation mutant S201D partially reversed phosphatase activity when compared with 201A (Figure 3D). Taken together, the results suggest that *O*-GlcNAcylation of PTP1B contributes to its phosphatase activity.

### 2.4. Intervention PTP1B O-GlcNAcylation Can Recover Insulin Sensitivity and Improve Lipid Metabolism

**Figure 4:**
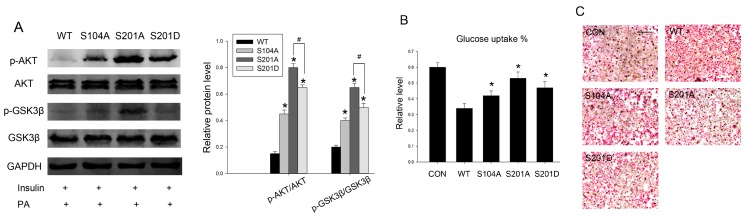
Intervention PTP1B *O*-GlcNAcylation can recover insulin sensitivity and improve lipid metabolism. Cells were first transfected with wild-type and different site-directed mutations and treated with 0.2 mM palmitate, then incubated for 20 min with 100 nM insulin. (**A**) The protein levels and phosphorylation of Akt and GSK3β were measured by western blot analysis; (**B**) 50 μL of medium was sampled for measurement of glucose concentration using a Glucose Colorimetric/Fluorometric Assay Kit. The primary medium was used as the control; (**C**) Lipid deposition was determined by Oil red O staining. Scale bars 50 μm. Data show mean ± SD of three independent experiments. * and ^#^ mean *p* < 0.05, significantly different from respective controls.

The relationship between PTP1B *O*-GlcNAcylation and its phosphatase activity in HepG2 cells warranted further investigation as to whether *O*-GlcNAcylation-deficient PTP1B can recover insulin sensitivity in palmitate-induced insulin resistance. To test this hypothesis, we transfected HepG2 cells with wild-type PTP1B and its mutants, then detected proximal steps in the insulin signaling pathway. We found that a deficiency in PTP1B *O*-GlcNAcylation resulted in an increase in insulin-stimulated phosphorylation of Akt and GSK3β, and hence might reverse the downstream events of insulin signaling. Accordingly, the dephosphorylation effect of PTP1B was in the same pattern with its phosphatase activity. The protective effect was more notable in S201A. Constitutive phosphorylation mutant S201D partially reversed the S201A protective effect (Figure 4A).

Impaired glucose uptake and abnormal lipid deposition are characteristics of insulin resistance [24], so we next examined whether S104A and S201A could exhibit the same beneficial effects. We observed that site-directed mutants partially reversed the negative effects of PTP1B on insulin-stimulated glucose uptake when compared with the wild-type (Figure 4B), consistent with its phosphatase activity, further confirming that *O*-GlcNAcylation-deficient PTP1B results in an increased sensitivity to insulin. Then we determined the effects of PTP1B *O*-GlcNAcylation on lipid metabolism. Lipid deposition was also improved by overexpression of PTP1B site-directed mutants. These metabolic alterations were associated with reduced PTP1B phosphatase activity, which was likely due to decreased PTP1B *O*-GlcNAcylation (Figure 4C), as previously shown.

### 2.5. Discussion

The highly abundant post-translational modification *O*-GlcNAc has been proposed to be a nutrient sensor as the donor sugar, UDP-GlcNAc, receives input from multiple metabolic pathways. Many studies have linked *O*-GlcNAc to the etiology of insulin resistance [22,25]. In diabetes and hyperglycemic states, increased intracellular protein *O*-GlcNAc modification has been observed. In recent years, PTP1B has emerged as an important negative regulator of insulin signaling. The fact that whole-body PTP1B knockout mice exhibit improved systemic insulin sensitivity prompted us to gain better knowledge of its role in the development of insulin resistance. Besides, diverse post-translational modifications of PTP1B have been reported, including oxidation, nitrosylation, sulfyhydration, and phosphorylation, but there is no report about PTP1B *O*-GlcNAcylation. In the current study, we have shown that PTP1B is also subjected to *O*-GlcNAcylation, and *O*-GlcNAcylation of PTP1B is increased when insulin resistance occurs. So we evaluated the functional contribution of PTP1B *O*-GlcNAcylation to insulin resistance.

As a phosphatase, PTP1B can inhibit insulin-stimulated IR autophosphorylation and phosphorylation of IRS, Akt, and GSK3β, thereby terminating the insulin signaling. Thus normal insulin signal transduction is partly the result of accurate regulation of PTP1B phosphatase activity. Here we demonstrated that *O*-GlcNAcylation increased PTP1B phosphatase activity under insulin resistance conditions, therefore leading to impaired insulin signaling in HepG2 cells. PTP1B *O*-GlcNAc levels were elevated by high glucose and PUGNAc treatment, and was subsequently reduced by insulin administration, which was in agreement with the previous studies that insulin induces PTP1B tyrosine phosphorylation and decreases its activity in skeletal muscle and adipose [26,27]. These findings indicate that there is a Yin-Yang effect with PTP1B.

Next we determined PTP1B with three *O*-GlcNAc sites: Ser104, Ser201, and Ser386, two of which (Ser201 and Ser386) overlap with its phosphorylation sites. Because how phosphorylation regulates PTP1B phosphatase activity is unclear and simple site-directed mutations will be unable to distinguish between the biological functions mediated by either *O*-GlcNAcylation or phosphorylation, it is complex to investigate the function of PTP1B *O*-GlcNAcylation, despite the fact that the interplay between *O*-GlcNAcylation and phosphorylation has been reported in other proteins [28,29,30]. Considering that Ser104 and Ser201 are within the conserved phosphatase catalytic domain, we supposed that the two *O*-GlcNAc sites might be important for regulating PTP1B phosphatase activity and functions. So we constructed site-directed mutants to further investigate its role in insulin resistance: Ser104 and Ser201 were mutated to alanine to block *O*-GlcNAcylation, while Ser201 was also mutated to aspartic acid to mimic constitutive phosphorylation. All the mutants represented a decreased state of PTP1B *O*-GlcNAcylation when compared with the wild-type, displaying markedly reduced phosphatase activity, and in turn resulting in the significant recovery of normal insulin signal transduction. Importantly, the effect of S201A was more significant, and the level of its phosphatase activity was concomitant with its *O*-GlcNAcylation level. This is possibly because Ser201 residue is closer to the essential catalytic cysteine 215, which is located at the base of phosphatase’s active site cleft. Constitutive phosphorylation mutant S201D partially reversed its phosphatase activity when compared with S201A, suggesting that the crosstalk between *O*-GlcNAcylation and phosphorylation is more complex than the “yin-yang hypothesis,” and this interplay is not a simple binary model with reciprocal on or off status. However, we were unable to test this possibility due to the lack of available phospho-site-specific anti-PTP1B antibodies. We know that PTP1B is phosphorylated on tyrosine and serine residues, which can either enhance or attenuate its enzymatic activity [26,27], but how phosphorylation at Ser201 regulates its enzymatic activity is unknown, and this issue will require further study in light of the importance of PTP1B regulating its activity post-translation.

In summary, our results emphasize the importance of PTP1B in the control of hepatic insulin signaling and reveal that *O*-GlcNAcylation represents a novel regulation of PTP1B activity and functions. Inhibition of PTP1B *O*-GlcNAcylation can recover insulin sensitivity, facilitate hepatic glucose uptake, and improve lipid deposition. Numerous studies have reported the beneficial effects of PTP1B inhibitors in enhancing insulin sensitivity. However, structural homologies in the catalytic domain of PTP1B with other PTPs like leukocyte common antigen-related, CD45, SHP-2, and T-cell-PTP present a challenging task of high selectivity [4]. Thus, highly selective molecules directed at *O*-GlcNAc sites of PTP1B are expected. It may provide a potential therapeutic target for the treatment and prevention of diabetes.

## 3. Experimental Section

### 3.1. Antibodies and Reagents

Akt, phospho-Akt (Ser473), GSK3α/β, phospho-GSK3α/β (Ser21/9) antibodies were obtained from Cell Signaling (Beverly, MA, USA). *O*-GlcNAc antibodies (CTD110.6 and RL-2), *O*-(2-acetamido-2-deoxy-d-glucopyranosylidene), amino-*N*-phenylcarbamate (PUGNAc), anti-PTP1B, anti-myc, anti-GAPDH antibodies, palmitate acid, and Oil Red O were from Sigma (Histon, WA, USA). Lipofectamine 2000 was obtained from Invitrogen (Carlsbad, CA, USA).

### 3.2. Cell Culture and Treatments 

Human HepG2 cells were grown in 5 mmol/L (low glucose) or 25 mmol/L (high glucose) Dulbecco’s modified Eagle’s medium supplemented with 10% fetal calf serum at 37 °C with humidified air and CO_2_ (5%). Then the cells were treated with palmitate acid (0, 0.2 or 0.4 mmol/L) for 24 h. Palmitate acid was prepared by conjugating it with bovine serum albumin and diluting it to appropriate concentrations as previously reported [23]. For some experiments cells were co-treated with 100 μM PUGNAc (Toronto Research Chemicals, Toronto, ON, Canada) overnight. Insulin was added during the last 20 min of incubation.

### 3.3. Animal Treatments

Animal studies were conducted in accordance with the National Institutes of Health Animal Care and Use Guidelines. Male six-week-old C57BL/6J mice were housed with 12 h light/dark cycle with free access to water and given a normal diet (ND) or high fat diet (HFD) containing 50% carbohydrate, 20% protein, and 30% fat for 12 weeks. A glucose tolerance test was conducted two weeks before sacrifice. Mice were fasted overnight and then dosed orally with glucose (2 g/kg). The tail vein blood glucose level was measured at 0, 20, 40, 60, and 120 min after administration.

### 3.4. Protein Extraction and Western Blotting

Cells were lysed in M-PER mammalian protein extraction reagent containing protease inhibitor mixture and phosphatase inhibitor mixture (Sigma), and fractional protein was extracted according to the manufacturer’s instructions. The protein level was quantified using the Bradford procedure. Two hundred micrograms of whole cell extracts were separated by SDS-PAGE and transferred to an enhanced nitrocellulose membrane. The blots were blocked with 5% skim milk for 1 h, and incubated overnight at 4 °C with the primary antibody at the dilutions recommended by the supplier. The membrane was then washed with TBST (10 mMTris-HCl, pH 7.6, 150 mM NaCl, 0.1% Tween 20), and the secondary antibodies conjugated to horseradish peroxidase were incubated for 1 h at room temperature. The bands were visualized using an enhanced chemiluminescence system (ECL; Pierce Company, Woburn, MA, USA). ImageJ was used to analyze the densities of the bands.

### 3.5. Identification of O-GlcNAc Sites

Human HepG2 cells were treated with PUGNAc to inhibitβ-*N*-acetylglucosaminidase and lysed in 1.5% SDS containing Complete™ protease inhibitor cocktail (Roche, Basel, Switzerland) and 5 µM PUGNAc. The lysate (7.5 mg) was diluted 1.5-fold, quenched with one volume of NETFS buffer containing 6% (*v*/*v*) NP-40 and then was further diluted to 2 mg/mL with NETFS buffer. The sample was passed over 400 μL of anti-PTP1B M2 affinity gel three times, washed with 10 mL of NETFS containing 1% (*v*/*v*) NP-40, eluted in 400 μL of 4% SDS, 100 mM Tris separated pH 7.9, and concentrated to a volume of 20 μL. After SDS-PAGE (4-12% Bis-Tris gels), the PTP1B band was excised and manually digested in gel with trypsin or Glu-C at 37 °C. LC-MS analysis was carried out on LTQ mass spectrometer (Thermo, Waltham, MA, USA). Peptide mixtures were on a C18 column. Mass spectrometric data were searched by SEQUST against PTP1B protein sequence. The relevant searching parameters of mass range, intensity threshold, minimum ion count, precursor ion mass tolerance, and fragment ion mass tolerance were set as 500–5000, 500, 15, 3.0, and 1.0, respectively. Trypsin or Glu-C was designated as the protease, and up to two missed cleavages were allowed. Carbamidomethylation of Cys was a fixed modification, and phosphorylation (80.0 Da) and *O*-GlcNAcylation (203.2 Da) of Ser/Thr, and oxidation of Met (16 Da) were allowed as variable modifications.

### 3.6. Plasmid Constructs

Human wild-type PTP1B plasmid was obtained from Genechem (Shanghai, China), inserted into pcDNA3.1/myc-His (−) vector. Site-directed mutants (Ser104 mutated to alanine, Ser201 mutated to alanine and aspartic acid) were performed by PCR using mutagenic oligonucleotides using human wild-type PTP1B plasmid as template. The primers used in the mutations were the following: S104A, forward, 5-TGGGAGCAGAAAGCAAGGGGTGTCGT-3, and reverse, 5-ACGACACCCCTTGCTTTCTGCTCCCA-3, S201A, forward, 5-AAAGTCCGAGAGGCAGGGTCACTCAG-3, and reverse, 5-CTGAGTGACCCTGCCTCTCGGACTTT-3, S201D, forward, 5-AAAGTCCGAGAGGATGGGTCACTCAG-3, and reverse, 5-CTGAG TGACCCATCCTCTCGGACTTT-3. Mutagenized plasmids were checked by sequencing, and a region containing the mutation was systematically subcloned into the original pcDNA3.1/myc-His (−) vector using endogenous restriction sites to prevent mutations that may have occurred elsewhere on the plasmid.

### 3.7. Transfections

Wild-type PTP1B, different site-directed mutations plasmids were constructed as previously described. Using lipofectamine 2000, 5 mg of plasmid was transfected for 36 h in HepG2 cells according to the manufacturer.

### 3.8. In Vitro O-GlcNAc Modification Assay

Cells were transfected with myc-His-tagged full-length wild-type PTP1B or different point mutations. After 36 h, cells were collected in 50 mM Tris-Cl (pH 7.5), 150 mM NaCl, 1 mM EDTA, 1% Nonidet P-40 with protease inhibitors and then lysed by vortexing for 2 h. Equal amounts of lysate were incubated overnight with 5 μL of anti-*O*-GlcNAc antibodies rotating at 4 °C, followed by incubation with 30 μL of protein A/G PLUS-agarose (Santa Cruz Biotechnology, Santa Cruz, CA, USA) for 3 h at 4 °C. Immunoprecipitates were extensively washed, resuspended in 2× sample buffer, boiled for 15 min, and analyzed by immunoblotting. Another method to detect terminal GlcNAc-specific lectin is to add succinylated wheat germ agglutinin (WGA) agarose beads to cell lysate and analyze by western blot with PTP1B antibody.

### 3.9. In Vitro PTP1B Phosphatase Assay

To detect PTP1B phosphatase activity, HepG2 cells were transfected with wild-type PTP1B and its mutants for 36 h, followed by insulin stimulation for 20 min. Cells were lysed and 50 μL of different cell extracts were used as samples. *In vitro* PTP1B phosphatase assays were carried out according to the manufacturer’s instruction (SensoLyte^®^FDP Protein Phosphatase Assay Kit*Fluorimetric*, Frement, CA, USA).

### 3.10. Glucose Uptake Assay

HepG2 cells were transfected with wild-type PTP1B and its mutants for 36 h. Insulin was added during the last 20 min of incubation. A quantity of 50 μL of medium was sampled for measurement of glucose concentration using a Glucose Colorimetric/Fluorometric Assay Kit (BioVision, Milpitas, CA, USA). The primary medium was used as control. Glucose concentration was normalized with cellular protein concentration.

### 3.11. Oil Red O Staining

After transfection with myc-His-tagged wild-type PTP1B or different point mutations for 36 h, cells were washed three times with PBS and then fixed in 4% paraformaldehyde for 30 min and washed with 60% isopropyl alcohol. Then the cells were stained for 60 min in 0.2% Oil Red O dissolved in 60% isopropyl alcohol. Excess stain was removed by washing with 70% ethanol. The stained cells were finally washed three times with distilled water. Cells were imaged using a microscope.

### 3.12. Statistical Analyses

Data are expressed as the mean ± SEM of results of three or more independent experiments. Statistical analyses were performed using one-way ANOVA, followed by individual *post hoc* comparisons. The critical α level (*p*) was set at 0.05; *p* < 0.05 was considered statistically significant. GraphPad Prism 5 statistical software was used for analyses.

## 4. Conclusions

In conclusion, our findings show that PTP1B is an *O*-GlcNAc modified protein and *O*-GlcNAcylation of PTP1B is increased in insulin resistance. *O*-GlcNAc site-directed mutants can partially inhibit PTP1B phosphatase activity, thereby reversing insulin signal transduction, facilitating glucose uptake, and improving lipid metabolism.
